# Predictors of Frailty in the Elderly Population: A Cross-Sectional Study at a Tertiary Care Center

**DOI:** 10.7759/cureus.30557

**Published:** 2022-10-21

**Authors:** Ashwani Kumar, Minakshi Dhar, Mayank Agarwal, Anirudh Mukherjee, Vartika Saxena

**Affiliations:** 1 Internal Medicine, All India Institute of Medical Sciences, Rishikesh, Rishikesh, IND; 2 Internal Medicine, All India Institute of Medical Sciences, Raebareli, Raebareli, IND; 3 Community and Family Medicine, All India Institute of Medical Sciences, Rishikesh, Rishikesh, IND

**Keywords:** hills, urban, rural, frailty, predictors

## Abstract

Introduction

Frailty is a multidimensional complex state that leads to increased chances of hospitalization and death in patients, especially in the elderly. Our study aimed to determine the factors associated with the development of frailty and their predictors in the elderly population.

Methods

The study was conducted in the Outpatient Department (OPD) of General Medicine at a tertiary care hospital in Rishikesh town of Dehradun district, Uttarakhand, India. It was a cross-sectional study design, conducted from January 2019 to July 2020. Data regarding sociodemographic factors, medical conditions, and laboratory investigations were collected on a predesigned performa. Patients diagnosed with COVID-19 were excluded from the study. It being a hospital-based study, participants with one frailty criteria were considered as non-frail and those with two or more than two as frail.

Results

We enrolled 149 patients in our study, based on the inclusion and exclusion criteria. The mean age of the patients was 67.50+/-6.74 years. A total of 87 (58.38%) participants had a frailty score > 2. Region of residence, body mass index (BMI), albumin, transferrin saturation, ferritin, vitamin D3, sodium, calcium, creatinine, urea, hemoglobin, glycosylated hemoglobin (HbA1c), number of prescribed drugs, substance dependence, power grip strength (PGS), slow walking time (SWT), low physical activity (LPA), self-reported exhaustion (SRE), unintentional weight loss (UWL), and erythrocyte sedimentation rate (ESR) were independent significant predictors of frailty.

Conclusion

Various modifiable factors were found to be predictors of frailty in adults. Timely identification and necessary interventions of these risk factors can provide valuable information for future prevention of the progression of frailty in the elderly.

## Introduction

Frailty is a unique state of reduced physiological reserve and capacity. It is considered a geriatric giant but may occur at any age. Aging itself leads to a gradual decline in physiological functions, but frailty is the result of an accelerated decline in physiological conditions, leading to impaired homeostatic capacity [1,2]. It is defined as a multidimensional complex state associated with the impairment of multisystem functions, leading to an increased risk of hospitalization and death in older people [3,4]. Various reasons including genetic and environmental factors that regulate the differential expression of genes in cells in different environments have been attributed to contribute to frailty and may be critical in the aging phenomenon [5]. There is a demographic shift in population over the world, and India is no exception.

According to the 2011 census in India, the geriatric population (>60 years) was 8.6% and is expected to reach the highest in the world by 2050 [6]. A study by Biritwum et al. concluded that the prevalence of frailty (standardized age) was the highest in India (56.9%) as compared to other countries (China, Ghana, Mexico, Russia, and South Africa) [7,8]. Frailty leads to longer hospital stays and increased mortality in patients [9,10]. In another study from India by Yeolekar and Kalekar, frailty was found to increase the risk of disability and death, because of inadequate reserves to maintain homeostasis [11].

Several factors have been found to be associated with geriatric frailty. In developed countries, these factors include age, black race, female gender, cardiovascular disease, number of comorbidities, functional disability, poor self-rated health, depressive symptoms, body mass index (BMI), smoking, low schooling level, low income, poor nutrition, poor cognitive function, and alcohol consumption [12,13]. In developing and underdeveloped countries, the risk factors for frailty include age, female gender, low level of education, lower socioeconomic status, low physical activity, comorbidities, functional status, and nutritional status [14]. Studies have shown that factors associated with frailty are either preventable, such as malnutrition, BMI, and smoking habits; for some, the timely intervention of some factors, such as adequate control of comorbidities, can delay the onset of frailty [15].

Since only a handful of studies on the predictors of frailty have been conducted in India and possibly none reported from the state of Uttarakhand, we conducted a cross-sectional study at a tertiary care center, which is unique in its geographic location, as it caters to the population of both hills and plains. The main purpose of the study was to identify the factors associated with the development of frailty so that interventions can be planned at multiple levels while addressing the older population.

## Materials and methods

Study design

We conducted a cross-sectional study to assess the predictors of frailty among the elderly population aged 60 years and above attending the Outpatient Department (OPD) of General Medicine in a tertiary care hospital in Rishikesh, Dehradun district, Uttarakhand, India. The study was conducted after getting approval from the Institutional Ethics Committee (IEC) at All India Institute of Medical Sciences, Rishikesh (Letter Number AIIMS/IEC/19/1068). The study was conducted over a period of 18 months from January 2019 to July 2020.

Participants

A total of 149 patients were recruited based on the inclusion and exclusion criteria after taking informed consent by simple random sampling method.

Inclusion Criteria

We included all consenting adult patients aged 60 years and above attending the Geriatric OPD for routine follow-up visits.

Exclusion Criteria

Patients with acute illness/acute exacerbation on chronic condition, unable to perform walk tests due to physical disability (orthopedic deformity/disease, stroke, etc.), and with dementia, intellectual disability, or psychiatric illnesses were excluded.

Data collection

All patients were informed in detail about the purpose of the study, and after obtaining informed consent, the study was commenced. Information regarding demographic characteristics, comorbidities, and baseline hematological and biochemical parameters were collected on a predesigned performa. Frailty was assessed using Fried’s frailty criteria.

Fried’s frailty criteria

Fried’s frailty criteria were used to assess frailty [16]. It comprises five dimensions that are hypothesized to reflect systems whose impaired regulation underlies the syndrome. These five dimensions are shown in Table 1.

**Table 1:** Fried’s frailty criteria BMI: body mass index; GS: grip strength

Frailty criteria (present: 1, absent: 0)					
Unintentional weight loss of more than 4.5 kg in a year					
Exhaustion: feels exhausted most of the time or all the time for one month					
Muscle weakness (power grip strength (GS): average of three trials)					
For men			For women		
BMI (kg/m^2^)	GS (kg)		BMI (kg/m^2^)		GS (kg)
≤24	≤29		≤23		≤17
24.1-26	≤30		≥23.1-26		≤17.3
26.1-28	≤30		26.1-29		≤18
>28	≤32		>29		≤21
Slow walking speed time 6-meter walk					
For men				For women	
Height (cm)		Time to walk (seconds)		Height (cm)	Time to walk (seconds)
≤173		≥7		≤159	≥7
>173		≥6		>159	≥6
Low physical activity					
For men: <128 kcal/week					
For women: <90 kcal/week					

The stages of frailty, based on Fried’s criteria, were defined as follows: a score of 0 means that a person is robust or not frail, those with a score of 1 or 2 are at intermediate risk for adverse outcomes or are considered to be pre-frail, and a score of 3-5 indicates that the person is frail.

Statistical analysis

Data were entered in a Microsoft Excel spreadsheet (Microsoft Corp., Redmond, WA, USA), and analysis was done using Statistical Package for the Social Sciences (SPSS) version 23.0 (IBM SPSS Statistics, Armonk, NY, USA). Categorical variables were presented as numbers and percentages (%), and continuous variables were presented as means and standard deviations (SD), and median and interquartile ranges (IQR). The normality of data was tested using the Kolmogorov-Smirnov test, and where normality was rejected, a non-parametric test was used. Quantitative variables were compared using the independent t-test/Wilcoxon-Mann-Whitney test (if the datasets were not normally distributed) between two groups and the Kruskal-Wallis test between three groups. Qualitative variables were correlated using a chi-square test/Fisher’s exact test. A P value of <0.05 was considered statistically significant.

## Results

We enrolled 149 patients in our study, based on the inclusion and exclusion criteria. The mean age of the patients was 67.55 years (SD = 6.73; range = 62-72 years) with 91 (60.8%) males and 58 (39.2%) females. This being a hospital-based study, the participants were divided into two groups: frail and non-frail. For analysis purposes, the participants in the non-frail and pre-frail categories were categorized as non-frail, and those with scores of 3-5 were categorized as frail. Table 2 depicts that demographic factors, such as age, region of residence, and number of comorbidities, are significantly associated with frailty. Table 3 shows that hand grip was the most common frailty component affected in the study population.

**Table 2:** Association between frailty score, demographic parameters, and comorbidities

Parameter	Total (N=149)	Frailty score of 3-5 (n=87)	Frailty score of 0-2 (n=62)	P value	X^2^
Age (years)	67.50+/-6.74	68.35+/-7.04	65.35+/-6.15	0.265	-
Gender				0.09	2.75
Male	91 (61.07%)	58 (63.73%)	33 (36.26%)		
Female	58 (38.92%)	29 (50%)	29 (50%)		
Residence				0.64	0.20
Urban	73 (48.99%)	44 (60.27%)	29 (39.72%)		
Rural	76 (51.01%)	43 (56.58%)	33 (43.42%)		
Region				0.03	4.64
Plains	112 (75.17%)	71 (63.40%)	41 (36.60%)		
Hills	37 (24.83%)	16 (43.24%)	21 (56.76%)		
Socioeconomic status				0.38	0.76
Upper	47 (31.54%)	25 (53.19%)	22 (46.81%)		
Lower	102 (68.45%)	62 (60.78%)	40 (39.21%)		
Number of comorbidities				0.05	5.87
0	39 (26.17%)	17 (43.60%)	22 (46.80%)		
1	53 (35.57%)	31(58.49%)	22 (41.51%)		
≥2	57 (38.25%)	39 (68.42%)	18 (31.58%)		
Living arrangement				0.68	
With family	144 (96.64%)	85 (59.03%)	59 (40.97%)		
Alone	5 (3.34%)	2 (40%)	3 (60%)		
Spouse				0.74	0.11
Alive	129 (86.57%)	76 (58.91%)	53 (41.08%)		
Lost	20 (13.42%)	11 (55%)	9 (45%)		
Financial dependence				0.46	0.53
Self	115 (77.18%)	69 (60%)	46 (40%)		
Family	34 (22.81%)	18 (52.94%)	16 (47.06%)		
Substance dependence (present)	56 (37.58%)	43	13	>0.001	12.5
Number of prescription drugs				<0.001	10.09
0-3	78 (52.34%)	36 (46.15%)	42 (53.84%)		
>3	71 (46.97%)	51 (71.83%)	20 (28.17%)		

**Table 3:** Summary of frailty components in the study population (N=149) PGS: power grip strength; SWT: slow walking time; LPA: low physical activity; SRE: self-reported exhaustion; UWL: unintentional weight loss

Frailty components	Present (number (%))	Absent (number (%))
PGS score (decreased)	114 (77%)	34 (23%)
SWT	90 (60.8%)	58 (39.2%)
LPA	81 (54.7%)	67 (45.3%)
SRE	98 (66.2%)	50 (33.8%)
UWL	17 (11.5%)	131 (88.5%)

A total of 149 participants were included in the analysis for binomial logistic regression to assess the effect of various predictor variables on frailty. The binary outcome was considered as either “not frail” or “frail,” where the “not frail” category includes participants with Fried’s frailty scores between 0 and 2, and “frail” includes scores between 3 and 5. A total of 43 predictors were included in the forward stepwise conditional binary logistic regression analysis, which attributed to a 57.5% improvement in the model. Region of residence, BMI, albumin, transferrin saturation, ferritin, vitamin D3, sodium, calcium, creatinine, urea, hemoglobin, glycosylated hemoglobin (HbA1c), number of prescribed drugs, substance dependence, power grip strength (PGS), slow walking time (SWT), low physical activity (LPA), self-reported exhaustion (SRE), unintentional weight loss (UWL), and erythrocyte sedimentation rate (ESR) were independent significant predictors of frailty (Table 4). However, only two models that did not show multicollinearity were selected, and these models met the linearity assumption using model fit and pseudo-R-squared statistics.

**Table 4:** Association of frailty grade with biochemical parameters (N=149) ESR: erythrocyte sedimentation rate; HbA1c: glycosylated hemoglobin

Parameter	Frailty score of 3-5 (n=87)	Frailty score of 0-2 (n=62)	P value
Hemoglobin (g/dL)	10.33+/-2.33	12.33+/-1.92	<0.001
Platelet count (lac/mm^3^)	2.45+/-0.91	2.48+/-0.61	0.81
ESR (mm/hour)	30.34+/-19.33	23.89+/-13.91	0.02
Urea (mg/dL)	42.87+/-38.48	30.03+/-21.91	0.01
Serum creatinine (mg/dL)	1.4+/-1.79	0.77+/-0.46	<0.001
Serum sodium (mEq/L)	137.39+/-4.33	139.47+/-4.35	0.004
Serum potassium (mEq/L)	4.06+/-0.55	4.07+/-0.51	0.91
Serum calcium (mg/dL)	8.75+/-0.54	9.02+/-0.63	0.005
Uric acid (mg/dL)	5.86+/-5.3	4.65+/-1.39	0.04
Serum vitamin D3 (ng/dL)	31.55+/-11.19	37.15+/-10.52	0.002
HbA1c (%)	6.33+/-1.05	5.95+/-0.97	0.02
Serum iron (µg/dL)	60.64+/-29.28	79.72+/-25.57	<0.001
Serum ferritin (ng/mL)	83.25+/-77.65	108.95+/-47.44	0.01
Transferrin saturation (%)	28.02+/-11.06	35+/-8.37	<0.001
Serum albumin (mg/dL)	3.73+/-0.35	3.86+/-0.31	0.01
Serum B12 levels	313.46+/-140.89	303.93+/-113.92	0.66
Serum folate levels	6.17+/-2.68	5.78+/-2.09	0.34

Model 1

In model 1, the predictor variables were PGS in kg, SWT, LPA, and SRE (Table 5). There were no outliers reported. The logistic regression model was statistically significant (X^2^ (4)=150.511, P≤0.001). The model explained 64.3% and 86.4% (Cox-Snell and Nagelkerke R-square) of variance in frailty and correctly classified frail categories in 93.8% of cases. All predictors were statistically significant. Frailty decreased by 0.78 for a unit increase in PGS.

**Table 5:** Model 1 summary of binomial logistic regression of predictor variables for frailty PGS: power grip strength; SWT: slow walking time; LPA: low physical activity; SRE: self-reported exhaustion; CI: confidence interval

Variable	B	Wald	P value	Crude odds ratio with 95% CI	Adjusted odds ratio with 95% CI
PGS	-0.245	10.652	0.001	0.836 (0.78, 0.89)	0.783 (0.67, 0.91)
SWT	5.487	22.135	<0.001	26.4 (10.86, 64.15)	241.58 (24.56,2375.85)
LPA	4.747	17.392	<0.001	10.49 (4.87, 22.58)	115.22 (12.38, 1072.45)
SRE	5.291	17.490	<0.001	6.64 (3.14, 14.08)	198.50 (16.63, 2369.36)

Model 2

In model 2, the predictor variables were PGS in kg, SWT, LPA, SRE, and sex (Table 6). No outliers were found. The logistic regression model was statistically significant (X^2^ (5)=162.013, P≤0.001). The model explained 67% and 90.1% (Cox-Snell and Nagelkerke R-square) of variance in frailty and correctly classified 96.6% of cases. All predictors were statistically significant. Frailty decreased by 0.78 for a unit increase in PGS. Other predictors increased the odds of frailty with each unit increase in the predictor variable.

**Table 6:** Model 2 summary of binomial logistic regression of predictor variables for frailty PGS: power grip strength; SWT: slow walking time; LPA: low physical activity; SRE: self-reported exhaustion; CI: confidence interval

Variable	B	Wald	P value	Crude odds ratio with 95% CI	Adjusted odds ratio with 95% CI
PGS	-0.355	10.163	0.001	0.84 (0.78, 0.89)	0.70 (0.56, 0.87)
SWT	6.461	19.114	<0.001	26.4 (10.86, 64.15)	639.442 (35.31, 11578.23)
LPA	5.454	16.197	<0.001	10.49 (4.87, 22.58)	233.63 (16.4, 3326.83)
SRE	6.363	16.905	<0.001	6.64 (3.14, 14.08)	580.23 (27.93, 12050.44)
Sex (male)	3.129	7.817	0.005	1.75 (0.90, 3.43)	0.004 (2.54, 205.05)

## Discussion

We enrolled 149 participants based on inclusion and exclusion criteria and examined the proportion of frailty and its predictors in elderly patients. In our study, the proportion of frailty was 58.38%, consistent with a few other studies [7]. The proportion of pre-frailty was 36.5%. Region of residence, BMI, albumin, transferrin saturation, ferritin, vitamin D3, sodium, calcium, creatinine, urea, hemoglobin, HbA1c, number of prescribed drugs, substance dependence, PGS, SWT, LPA, SRE, UWL, and ESR were independent significant predictors of frailty. This might be the first study to analyze the predictors of frailty in Uttarakhand, India. The unique finding of this study was that participants belonging to hilly regions were found to have less frailty scores. Thus, area of residence came up as a new predictor of frailty.

Frailty increases with advancing age, but it did not come up as a predictor of frailty in our study. In men, the frailty/non-frailty ratio was 63.3:36.7, while in women, it was 50:50, depicting that men were frailer, which is contrary to other most similar studies [7]. This variability in our study is partially supported by the finding of Cohen et al [17]. They concluded that sex differences in frailty may relate to sex differences in physiological dysregulation patterns. According to them, there was no clear evidence for sex differences in rates of change in dysregulation with age. These findings imply that the greater susceptibility of women to frailty is not simply due to tolerance for higher dysregulation; rather, it may actually be men that have a greater tolerance for dysregulation, creating a male-female dysregulation-frailty paradox [17]. In most of the previous studies, the prevalence of frailty was higher in women than in men, except in studies from Mexico and rural China. In our study, women were more in the pre-frail group but less in the frail group compared to men, showing that conversion from pre-frailty to frailty is low in women in comparison to men. More focused studies are required to find the precise physiological mechanisms underlying the sex differences in frailty.

The proportion of participants from plains and hills was 75% and 25%, respectively. Our study showed that people living in hills have a lower frailty score as compared to those living in plains, indicating that the population from hills is robust and less frail compared to the population from plains. To the best of our knowledge, this may be the first study to find the relationship between the area of residence and frailty. Factors that may be contributory to this are less percentage of sarcopenic obesity among people hailing from hills. People from hills usually are more physically active, leading to better physical fitness and favorable BMI. This results in frailty-free survival in the hilly population.

Substance dependence was significantly associated with frailty, and this was a predictor of frailty. Substance dependence was more in participants coming from hills compared to plains. Polypharmacy is another challenge and was found to be a strong predictor of frailty. Results from previous studies show that polypharmacy is 51% in pre-frail and a higher probability of up to 74% in frail [18]. In our study, polypharmacy (>3 prescription drugs) was present in 46.97% of the total participants. Of the participants belonging to the non-frail and pre-frail categories as per Fried’s frailty score, 28.17% were consuming >3 prescription drugs, whereas 71.83% of frail participants were consuming >3 prescription drugs. These findings were comparable to other studies conducted by Rakesh et al. [19], Veronese et al. [20], and Chen et al. [21]. This finding emphasizes that there is a strong need to create awareness among healthcare professionals toward their drug-prescribing behavior for the elderly to prevent polypharmacy and reduce the pill burden.

There was no association of socioeconomic status with frailty. One of its possible reason may be the growing trend of increasing BMI and sedentary lifestyles among all social groups that nullify the health benefit in the affluent class [8].

Biochemical parameters such as hemoglobin level, iron, ferritin, urea, creatine, and albumin were found to be predictors of frailty in the elderly in our study.

A decrease in the level of circulating hemoglobin [22], albumin [23], and low glomerular filtration rate [24] have also been shown by several authors to correlate with the incidence of frailty. These markers also correlate with the key symptoms of frailty, such as exhaustion and muscle weakness. Identification and treatment of the biochemical parameters at an early stage will help in delaying the onset of frailty in the elderly.

Glucose dysregulation has been found to be associated with frailty. HbA1c can be easily measured and is coming up as a promising marker of frailty [25]. However, lower HbA1c has also been associated with an increased incidence of falls in the elderly, confusing the picture of the relationship between glycemic control and outcomes in these patients [26].

Our study showed that lower levels were a predictor of frailty in the elderly population. Increasing sedentary habits among the elderly predispose them to vitamin D deficiency. Low levels of vitamin D have been shown to correlate with increased rates of frailty, as well as correlating strongly with the transition into frailty and pre-frailty in various other studies as well [27]. Studies have shown that vitamin D supplementation could cause a reversal from pre-frail to robust, but not from frail to pre-frail, providing some evidence of a mechanistic relationship between the two [28].

Limitation

As the study was conducted during the COVID-19 pandemic, the estimation of frailty may not be accurate, although we excluded all those participants who had symptoms suggestive of COVID-19.

## Conclusions

The proportion of frailty in participants coming from hilly areas was low compared to plains (43.24% versus 63.39%). We also concluded that females are more pre-frail, but males are frailer.

Early identification of risk factors of frailty can provide us valuable information for future prevention of progression and timely intervention. We observed that low vitamin D and iron levels were predictors of frailty and should be investigated and treated early to halt the progression of frailty from pre-frailty. Smoking was found to be an important contributor to frailty and is more common in inhabitants of hilly areas.

We recommend more studies at the national level to establish the strength of the relationship between modifiable risk factors and frailty to provide a better quality of life to our aging population. Our findings suggest that community-based awareness programs for the promotion of physical activity as important components in urban areas to reduce frailty and should be incorporated into central/state government urban health programs. The government could focus on screening frailty at the primary and community health center level to delay an irreversible but avoidable disabling process.
